# The Effects of Aerobic‐Strength Training on Cognitive Functions and Residual Platinum Levels in Cancer Survivors

**DOI:** 10.1002/alz.089575

**Published:** 2025-01-09

**Authors:** Barbara Ukropcová, Ali Amiri, Lucia Slobodova, Karin Marček Malenovská, Radka Pechancova, Katarina Rerkova, Martin Schön, Viktor Oliva, Viera Litvakova, Milan Sedliak, Martin Krssak, Tomas Pluhacek, Michal Mego, Michal Chovanec, Jozef Ukropec

**Affiliations:** ^1^ Biomedical Research Center, Slovak Academy of Sciences, Bratislava Slovakia; ^2^ Institute of Pathophysiology, Faculty of Medicine, Comenius University, Bratislava Slovakia; ^3^ Faculty of Science, Palacky University Olomouc,, Olomouc Czech Republic; ^4^ Faculty of Physical Education and Sports, Comenius University, Bratislava Slovakia; ^5^ High‐Field MR Centre, Medical University of Vienna, Vienna Austria; ^6^ National Cancer Institute, and Faculty of Medicine, Comenius University, Bratislava Slovakia

## Abstract

**Background:**

The risk of cognitive decline in cancer survivors may be increased by platinum‐based chemotherapy. Evidence indicates that physical exercise has a potential to reduce chemotherapy‐related toxicity. The aim of this study was to assess effects of a 6‐month aerobic‐strength training on cognitive functions, metabolic flexibility, anthropometric parameters and physical fitness in testicular germ cell tumor (TGCT) survivors, treated with platinum‐based chemotherapy.

**Methods:**

TGCT survivors underwent 6‐month intervention (supervised aerobic‐strength training/ controls n = 20/8, age 42.1±7.6 yrs. BMI:27.7±3.8kg/m², VO_2_max:29.2±6.7 mlO_2_/kg/min, 1‐20 years post‐treatment). Cognitive functions (FACT‐Cog; computerized tests Memtrax & CogState; Auditory Verbal Learning Test/AVLT), abdominal adiposity (MRI), cardiorespiratory fitness (VO_2_max, spiroergometry), muscle strength (leg‐press; chair dynamometry), energy expenditure and metabolic substrate preference (indirect calorimetry), serum glycemia, insulinemia, lipid profile, plasma adiponectin (ELISA), platinum, and zinc (Inductively‐Coupled Plasma Mass Spectrometry) were assessed before and after the 6‐month intervention.

**Results:**

Training improved delayed and short‐term memory recall (AVLT), compared to non‐active controls. Training‐induced changes in delayed memory recall positively correlated with improvements in VO_2_max, supporting a link between cognition and cardiorespiratory fitness. Training reduced visceral adiposity (p<0.01), increased HDL‐cholesterol (p<0.01), cardiometabolic fitness (VO_2_max) and muscle strength (both p = 0.01). Plasma adiponectin, glycemia and insulinemia were unaffected by exercise training (p>0.1), while intervention normalized residual plasma platinum and increased zinc levels (both p<0.01), with no change in controls. Platinum levels (P<0.001) and number of chemotherapy cycles (p = 0.06) were the best predictors of memory function in cancer survivors.

**Conclusions:**

Aerobic‐strength exercise training reduced visceral adiposity and circulating residual levels of platinum, improved physical fitness and cognitive functions in TGCT cancer survivors. Our observations support a role for exercise as an effective complementary treatment for cancer survivors, with a potential to reduce residual chemotherapy burden and cardiometabolic risk, which could be linked to improved cognitive functions.

**Funding**: APVV 19‐0411, VEGA2/0164/20, VEGA 2/0076/22

